# Potential Broad Spectrum Inhibitors of the Coronavirus 3CL^pro^: A Virtual Screening and Structure-Based Drug Design Study

**DOI:** 10.3390/v7122963

**Published:** 2015-12-15

**Authors:** Michael Berry, Burtram C. Fielding, Junaid Gamieldien

**Affiliations:** 1 South African Medical Research Council Bioinformatics Capacity Development Unit, South African National Bioinformatics Institute, University of the Western Cape, Bellville 7535, South Africa; michael@sanbi.ac.za (M.B.); junaid@sanbi.ac.za (J.G.); 2 Molecular Biology and Virology Laboratory, Department of Medical BioSciences, Faculty of Natural Sciences, University of the Western Cape, Western Cape, Bellville 7535, South Africa

**Keywords:** human coronaviruses, molecular docking, 3CL^pro^, virtual screening, structure-based drug design, molecular dynamics

## Abstract

Human coronaviruses represent a significant disease burden; however, there is currently no antiviral strategy to combat infection. The outbreak of severe acute respiratory syndrome (SARS) in 2003 and Middle East respiratory syndrome (MERS) less than 10 years later demonstrates the potential of coronaviruses to cross species boundaries and further highlights the importance of identifying novel lead compounds with broad spectrum activity. The coronavirus 3CL^pro^ provides a highly validated drug target and as there is a high degree of sequence homology and conservation in main chain architecture the design of broad spectrum inhibitors is viable. The ZINC drugs-now library was screened in a consensus high-throughput pharmacophore modeling and molecular docking approach by Vina, Glide, GOLD and MM-GBSA. Molecular dynamics further confirmed results obtained from structure-based techniques. A highly defined hit-list of 19 compounds was identified by the structure-based drug design methodologies. As these compounds were extensively validated by a consensus approach and by molecular dynamics, the likelihood that at least one of these compounds is bioactive is excellent. Additionally, the compounds segregate into 15 significantly dissimilar (*p* < 0.05) clusters based on shape and features, which represent valuable scaffolds that can be used as a basis for future anti-coronaviral inhibitor discovery experiments. Importantly though, the enriched subset of 19 compounds identified from the larger library has to be validated experimentally.

## 1. Introduction

Coronaviruses (CoV) affect a diverse group of animal hosts, and cause a plethora of diseases in animals including progressive peritonitis, acute and chronic hepatitis, gastroenteritis, nephritis, and encephalitis [1]. In humans, coronavirus infection results in respiratory tract complications with varying degree of severity and has been associated with gastroenteritis. Four human coronaviruses (HCoV; 229E, OC43, NL63 and HKU1) are endemic in the human population and are mainly associated with mild, self-limiting respiratory illnesses. Another two human coronaviruses, namely severe acute respiratory syndrome (SARS)-CoV and Middle East respiratory syndrome (MERS)-CoV, cause severe respiratory syndromes and present a significant threat with their high fatality rates. The clinical presentation of the four non-severe, endemic, human coronaviruses is largely indistinguishable symptomatically, commonly presenting with rhinorrhea, sore throat, cough and fever [2,3]. Majority of infections are associated with self-limiting upper respiratory tract disease or “the common cold” but can also present with high morbidity outcomes of the lower respiratory tract including bronchiolitis, pneumonia, [4,5,6], asthmatic exacerbations [7] acute exacerbations of chronic obstructive pulmonary disease (COPD) [8] and croup in HCoV-NL63 infected patients [9], and commonly results in hospitalization. Febrile seizures have been reported for most human coronaviruses but the significance of HKU1 is alarming with one study indicating that 50% of patients infected with HKU1 experience febrile seizures [7].

HCoVs affect all age groups [2,3] but elicit more serious disease in young, elderly and immunocompromised [4,10,11], frequently resulting in hospitalization. Reports on the prevalence of HCoVs and their association with upper and lower respiratory tract disease vary but range between 3.3% and 16% [2,3,10,12]. Over 70% of the general public has seroconverted towards all four non-severe HCoVs with primary infection shown to occur in childhood [13] and reinfection occurring throughout life [10]. Given the high prevalence of respiratory infections, human coronaviruses represent a substantial disease burden, which is exacerbated by the high implications of healthcare workers in coronavirus outbreaks [12].

The outbreak of SARS-CoV in 2003 and MERS-CoV less than 10 years later highlights an additional significant threat of coronaviruses to humans and confirms that the SARS outbreak in 2003 was not an isolated incident. With the ever increasing diversity of animal coronavirus species, especially within bats, the likelihood of recombination leading to future outbreaks is high and the threat of potential pandemics is real as highly pathogenic coronaviruses continue to spill over from zoonotic sources into the human population. Misdiagnosis of these outbreaks pose a further substantial threat to healthcare workers with nosocomial spread to other patients putting further pressure on an already strained healthcare system. As there is currently no antiviral strategy to combat infection with human coronaviruses [14], and supportive care is the primary treatment regime, the importance of identifying broad spectrum lead compounds is increasing.

The 3CL^pro^ has proven to be a valuable target in drug discovery efforts and has been validated as an effective drug target in several studies. It has even been termed “the Achilles’ heel of coronaviruses” [15]. A wide variety of inhibitor classes, including peptidomimetic analogues, covalent inhibitors and small molecule inhibitors have been assessed. These compounds have been identified by *ab initio* structure-based design, high throughput and virtual screening [15,16], where inhibitors either target the enzyme active site or the allosteric dimerization domain [17,18]. The first generation of 3CL^pro^ inhibitors were irreversible peptidomimetic structures, often five residues in length with at reactive warhead at the terminus that formed a covalent bond between the thiolate anion of the catalytic Cys145 residue and the reactive atom of the warhead [19]. These reactive warheads have included Michael acceptors [20,21,22], aldehydes [23], epoxy-ketones [24], halo-methyl ketones [25], and trifluoromethyl ketones [26]. Peptide derivative warhead inhibitors were later followed by the development of non-peptidic covalent inhibitors [27,28]. The use of covalent inhibitors is however limited due there propensity for off-target side-effects and toxicity [29]. Recent studies have therefore focused more on the development of noncovalent inhibitors, which have generally produced large peptidomimetic compounds with low ligand efficiency [19] and currently there is still no effective therapy for the treatment of HCoVs [14]. All coronavirus 3CL^pro^ share a high sequence homology, as well as main chain architecture and substrate conservation [30,31], which makes the identification of broad spectrum lead compounds more viable.

The substrate binding site of the 3CL^pro^ has two deeply buried S_1_ and S_2_ subsites, as well as shallow S_1′_, S_3_ and S_4_ subsites with varying degrees of solvent exposure. Substrate specificity of coronavirus 3CL^pro^ is mainly determined by the P_1_, P_2_ and P_1′_ positions [31]. The P_1_ position has an absolute specificity for glutamine which stabilizes the S_1_ subsite via a hydrogen bond with the imidazole Nε2 of His162/3 and van der Waals interactions with surrounding residues of the S_1_ pocket. The P_2_ site has a preference for leucine or methionine to fill the hydrophobic S_2_ pocket. The sidechains of the S_3_ site are solvent-exposed and therefore this site is expected to tolerate a wide range of functionality, but shows a preference for basic residues [32]. Sidechains and backbones of residues surrounding the S_4_ site create a highly congested pocket which favors a small, hydrophobic residue in the P_4_ position, either Ser, Thr, Val or Pro [32,33,34]. The S_1′_ and S_2′_ subsites also accommodate small residues in the P_1′_ and P_2′_ positions, which may include Ser, Ala or Gly [33,35]. A typical cleavage recognition site is therefore (Ser, Ala)-(Val, Thr)-Leu-Glu ↓ (Ser, Ala, Gly), which is conserved among all coronavirus 3CL^pro^ [36]. These features can therefore be exploited in the design of potential broad spectrum lead compounds.

## 2. Materials and Methods

### 2.1. Consensus Docking and Scoring with Vina, Glide, Gold and Molecular Mechanics-Generalized Born Surface Area (MM-GBSA)

Crystal structures of the 3CL^pro^ for 229E, NL63, HKU1 and SARS-CoV were obtained from PDB. The 3CL^pro^ of OC43 was obtained by homology modeling [37]. Vina performed the initial high throughput screen of the Drugs-Now dataset from ZINC, comprising a total of approximately 6.5 million compounds. Ligand files were obtained in 3D SDF format and converted to PDB with Open Babel [38]. Ligand PDB files were then converted to PDBQT with the *prepare_ligand4.py* script.

The initial virtual screen was conducted with 2ZU2 (3CL^pro^ of HCoV-229E) as the receptor as it comprises the largest binding pocket and therefore should identify the largest amount of primary leads. The bound ligands of apo structures were removed to create an appropriate cavity within the binding site. For docking with Vina, the receptor was processed with Autodock Tools [39] where water molecules were removed and polar hydrogens and Gasteiger charges were added. Histidine was protonated in the neutral state at the epsilon nitrogen (Nε2) in accordance with crystal data. The grid box for searching was centered on the active His41 residue with grid box spacing set to 1 Å and extended 26 grid points in each direction to encompass the entire binding pocket. The top ranked 1500 ligands from the virtual screen formed the primary hit-list as this was an acceptable quantity for the medium throughput analysis and subsequent refinement. The lowest binding score within the hit-list corresponded to a binding affinity of −9.5 kcal/mol and therefore all compounds with an equivalent binding affinity were progressed.

For docking and later refinement with Glide the 3CL^pro^ structures were prepared in Maestro with Schrödinger’s Protein Preparation Wizard. Receptors were minimized by a restrained minimization using the OPLS2005 force field [40,41], during which, heavy atoms were restrained to remain within 0.3 Å of the original structure. To define the binding site for docking with Glide, each structure was aligned and an active inhibitor (obtained from the crystal structure of the HKU1 3CL^pro^) was superimposed over the active site. For docking in Glide the receptor grid was generated around the superimposed ligand to accurately encompass the binding pocket. The grid box was generated 10 Å in each direction and a constraint was set for the ligand to form a hydrogen bond with the pivotal His162 residue of the S_1_ active pocket. All other settings remained default to Glide receptor grid generation. Glide docking was performed with default settings using standard precision (SP) mode with the fulfilment of the previously defined constraint. Ligands which did not fulfil this constraint were automatically excluded. A binding score cut-off, defined as −7 kcal/mol, was used to further filter the hit-list. This cut-off was predetermined based on the binding affinity of a known inhibitor.

Filtered compounds were subsequently imported into GOLD for redocking into the 2ZU2 structure prepared in Maestro where the active site was again defined by superimposition of the active ligand. An automated docking procedure with default parameters were used including 50 docking attempts and clusters defined by heavy atoms of ligands separated by >0.75 Å root-mean-square deviation (RMSD). The GOLDScore fitness scoring function was used to rank ligands based on a GOLDScore cut-off of 60 [42]. To ensure an accurate prediction of binding pose, the RMSD between the poses predicted by Vina, Glide and GOLD were measured using the *rmsd.py* script of Schrödinger. Ligands where the RMSD was greater than 2 Å between predicted poses were excluded.

The Prime/MM-GBSA method was used to calculate the binding free energy of each ligand within the docking complex, where the pose predicted by Glide was utilized. The free energy of binding is the calculated difference between the minimized receptor-inhibitor complex and the sum of the energies of the minimized unbound inhibitor and receptor. Ligand poses were minimized using the local optimization feature in Prime, where energies of the complex were calculated with the OPLS-2005 force field and GBSA continuum solvent model. As a final filter a binding free energy cut-off of −80 kcal/mol was defined by determining the free energy of binding of a known active inhibitor.

### 2.2. Pharmacophore Model for VS

Pharmacophore tools of Molecular Operating Environment (MOE) [43] were used to define 3D pharmacophoric features using the 3IH ligand and 3D23 structure for the initial high throughput virtual screen. Features included an essential hydrogen bond with the His163 residue and a hydrophobic moiety in the P_2_ position. Excluded volumes of 1 Å were set to better consider the steric effects. Non-essential features included a hydrogen bond donor and acceptor in the Glu166 position, a hydrogen bond acceptor in the P_4_ pocket, a single hydrogen bond donor in the P_2_ and P_3_ pocket and an acceptor in the P_1′_ pocket. A total of four pharmacophoric features were required within the excluded volumes of 1 Å. The pharmacophore model again screened the Drugs-Now dataset. Ligands satisfying the pharmacophore query within an RMSD of 0.5 Å were protonated with Open Babel and steric clashes caused by the ligand in order to satisfy pharmacophore constraints in the absence of explicit hydrogens were removed by refine only docking with Glide. A hydrogen bond constraint with His163 was used as a primary filter. Compounds were immediately assessed with the MM-GBSA scoring function, to avoid possible false negatives of the less reliable docking scoring functions, where the previously described MM-GBSA cut-off of −80 kcal/mol was again implemented.

### 2.3. Characterization of Broad Spectrum Potential

The above mentioned techniques conducted a high throughput virtual screen of the ZINC Drugs-Now dataset by docking and pharmacophore modeling. The structure-based screen identified potential binders of 2ZU2, where the ligand-based approach focused on 3D23. To identify which of these compounds would potentially form broad spectrum inhibitors the remaining three 3CL^pro^ structures were incorporated into the study. All five structures were aligned and ligands identified by docking and pharmacophore modeling were superimposed over individual active sites. Side chains of residues in the active pocket assuming orientations that resulted in a steric clash with the ligand were refined and the ligand was minimized with Prime to better fit the pocket. MM-GBSA methods described previously were again applied to predict the free binding energy of the complex. As before, a binding energy of −80 kcal/mol was used as a cut-off and the hydrogen bond constraint in the S_1_ pocket was again applied to discriminate between potential binders and non-binders. In an effort to not miss potential leads the interpretation of certain parameters were left flexible if other parameters supported the advancement of the compound.

### 2.4. Molecular Dynamic Simulation

The CHARMM27 all atom force field was assigned to receptor structures using the three point TIP3P water model. All input hydrogens in the coordinate file were ignored and were assigned according to the force field. Histidine protonation states were assigned to the epsilon nitrogen in the neutral form in accordance with crystallographic data. The ligand, in the pose predicted by molecular docking, was prepared by SwisParam [44]. The protein-ligand complex was then placed in the center of a solvated, cubic box, 1 nm from the box edge. The system was solvated with the spc216 water model and sodium counter ions were added to neutralize the system. The system was energy minimized by a brief dynamics simulation using Steepest Descent Minimization until steepest descent converged to Fmax <1000. Long range electrostatic interactions were calculated with the Particle Mesh Ewald (PME) method [45] with a cutoff of 1 nm and periodic boundary conditions were applied. Both NVT and NPT equilibrations were run for 50,000 steps or 100 ps using a 2 fs time step and a leap-frog integrator. All bonds were constrained by the LINCS algorithm. PME was again used for long range electrostatics with a 0.16 Fourier spacing. Short range electrostatic and van der Waals cutoffs were 1.0 nm and 1.4 nm respectively. Coordinates, velocities and energies were saved every 0.2 ps. During NVT equilibration temperature coupling was achieved by V-rescale algorithm with a target temperature of 300 K. Pressure coupling during NPT equilibration was achieved via the Parrinello-Rahman algorithm and is incorporated in the NPT equilibration once temperature is stable to ensure the proper density is reached (approximately 1000 kg/m^3^). Following NVT and NPT system equilibration an unrestrained, 10 ns simulation (5,000,000 steps) was run with the leap frog integrator, saving coordinates, velocities and energies every 2 ps. All other parameters were maintained from the NVT and NPT equilibration including both temperature and pressure coupling.

## 3. Results

### 3.1. Starting Structures

The 3CL^pro^ of 229E was previously solved in complex with an inhibitor by X-ray crystallography at a high resolution of 1.8 Å [46]. The corresponding crystallographic structure, with Protein Data Bank (PDB) code 2ZU2, shows a dimeric structure with the inhibitor, zinc *N*-ethyl-*N*-phenyldithiocarbamate (EPDTC), coordinated to the catalytic dyad residues of His41 and Cys144 with a zinc-centered tetrahedral geometry. The apo form of the NL63 3CL^pro^ was solved in its dimeric form by X-ray crystallography to a high resolution of 1.6 Å with the respective PDB code, 3TLO. The HCoV-NL63 3CL^pro^ structure (3TLO) however remains unpublished in any academic journals. Recently, our group solved and published the 3CL^pro^ structure of OC43 by homology modeling [37].

The PDB code, 3D23, represents the structure of the 3CL^pro^ of HKU1. It was previously solved by X-ray crystallography to a resolution of 2.5 Å in complex with a peptidomimetic, covalent inhibitor [30]. The PDB entry contains a total of four chains, two chains form the active dimeric structure with the remaining two found in monomeric forms. Each chain contains the covalent inhibitor bound to the active site which undergoes nucleophilic attack of the active cysteine Sγ to form an irreversible, covalent bond. As this inhibitor possesses several pharmacophoric features common to the natural substrate and favorable to drug development it was utilized to develop a pharmacophore model for virtual screening.

There are several structures available which represent the SARS-CoV 3CL^pro^. However, the majority of these include structures in complex with a peptidomimetic, covalent inhibitor [46,47,48] or mutational studies [49,50,51]. At the time this study commenced, only one structure could be identified in complex with a small molecule inhibitor. This structure, with PDB code 3V3M [19], also represented the only coronavirus 3CL^pro^ with a noncovalent inhibitor. As this structure could be used for necessary benchmarking in latter studies, it was chosen to represent the SARS-CoV 3CL^pro^. The crystallographic structure contains a noncovalent, furyl amide ligand, ML188, which was identified by a high throughput screen, with subsequent lead optimization based on structure-activity relationships within the S_1_, S_1′_ and S_2_ binding pockets.

### 3.2. Defining Benchmarks and Determining Accuracy of Vina and Glide

Molecular docking algorithms are often calibrated on a training set of experimental ligand-protein complexes and accuracy of these docking programs is often highly dependent on the training set used [52]. This highlights the importance of confirming that the docking software used for virtual screening is capable of replicating the binding mode of a known, experimental inhibitor for the class of enzymes studied [53,54].

In order to confirm that the molecular docking algorithms used in this study are well suited to the 3CL^pro^ of human coronaviruses, and their related class, a previously solved experimental structure with a bound, noncovalent inhibitor is required. Accurate replication of the experimental binding pose by molecular docking will confirm the suitability of the docking algorithm for virtual screening. Initial studies on structure-based design of inhibitors, targeting the 3CL^pro^ of coronaviruses, were focused on peptidomimetic compounds with a reactive warhead group that formed an irreversible, covalent interaction with the active Cys residue [19]. These large, covalent inhibitors would be incompatible with molecular docking as replication of the quantum mechanics necessary for covalent bond formation is outside the capabilities of molecular docking [55]. From the PDB several experimental structures of the 3CL^pro^ of various species of coronaviruses with bound inhibitor could be identified. Almost all of these structures represented large, peptidomimetic, covalent inhibitors, with the exception of 3V3M [19]. The structure of 3V3M represented the only noncovalent inhibitor of modest size that we could identify from the PDB. Replication of the binding mode of the bound inhibitor, ML188, by the docking algorithms used in the virtual screen and subsequent refinement process would indicate that they are well calibrated for this class of receptor.

**Figure 1:**
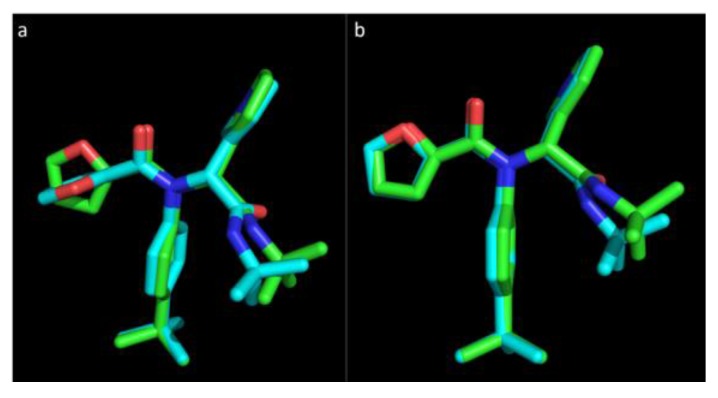
The pose of an experimental ligand, ML188, as predicted by Vina and Glide. (**a**) Experimental orientation of ML188 (**green**) with overlaid pose predicted by Vina (**blue**); (**b**) Experimental orientation of ML188 (**green**) with overlaid pose predicted by Glide (**blue**). An root-mean-square deviation (RMSD) of 0.244 Å for Vina and 0.79 Å for Glide suggests that they are well calibrated for this class of enzyme and should achieve a high level of accuracy in predicting the pose of novel ligands.

Default parameters applied to the high throughput virtual screen for Vina and refinement with Glide were applied to the inhibitor and receptor of 3V3M. An RMSD below 2 Å for heavy atoms (excluding hydrogens) between the experimental structure and predicted pose of docking is a well-defined benchmark to assess the accuracy of molecular docking sampling algorithms [56]. Figure 1 represents the experimental pose of the ML188 ligand with overlaid predicted pose of Vina and Glide.

Vina was able to accurately predict the pose of ML188, where the top ranked pose was within an RMSD of only 0.244 Å to the experimental structure within the pocket of 3V3M (Figure 1a). The orientation of the P_1′_ furan is somewhat distorted with the conformation of the ring structure being assumed to orientate at a right angle to the experimental pose. The overall predicted pose of Vina corresponds to the experimental pose with a high level of accuracy. Glide was also capable of accurately predicting the pose of ML188 within the pocket of 3V3M within an RMSD of 0.79 Å (Figure 1b). This indicates that sampling by both Vina and Glide are well calibrated for the class of enzyme studied and, as the remaining structures are highly homologous to 3V3M, they will achieve an equivalent level of accuracy. These results strongly support the use of Vina as high throughput tool for virtual screening and indicates that with the use of Glide as a subsequent refinement tool, the accuracy of predicting the binding pose of novel ligands will be high.

The ML188 lead compound also provided a means to set benchmarks for scoring functions and free energy of binding estimation. The inhibitor achieved a binding affinity estimation of −8.1 kcal/mol when bound to 3V3M. In an effort to reduce the numbers of false negatives this value was decreased to −7 kcal/mol for filtering purposes. For MM-GBSA scoring it is advisable that the threshold value is defined by an experimental structure [57]. The highly homologous structure of 3V3M with the known active inhibitor ML188 was therefore scored with MM-GBSA. The free energy of binding between 3V3M and ML188, where ligand strain was taken into account, was equal to −85 kcal/mol. As this ligand is highly suited towards the structure of 3V3M, where our goal is the identification of broad spectrum inhibitors, the cut-off value was decreased to −80 kcal/mol. This was done to ensure that ligands with potential broad spectrum activity were not missed based on their predicted inability to bind to a single receptor.

### 3.3. Molecular Docking

Vina was chosen as the tool to conduct the initial high throughput screen of the approximately 6.5 million compounds of the Drugs-Now dataset of the ZINC database. For the initial screen, 2ZU2 was selected as the receptor to identify the highly refined subset of potential inhibitors. This receptor possessed the largest binding pocket of all structures when analyzed by SiteMap (data not shown) and it was therefore proposed to provide the largest number of initial hit compounds. The primary hit-list consisted of the top 1500 ranked compounds from the high throughput virtual screen (HTVS) of the ZINC Drugs-Now dataset conducted by Vina, a quantity where further refinement and analysis would be achievable. Ranking the initial virtual screen based on docking score alone serves as a starting point in the selection criteria and assumes that the predicted binding affinity somewhat reflects true binding affinity [58,59,60]. The lowest binding score within this hit-list corresponded to −9.5 kcal/mol. Scoring functions have shown success in differentiating between binders and non-binders [61] by ranking the binding score. For this reason, all ligands which achieved this score were also included in the study, yielding a total of 1527 hit compounds. Further assessment and refinement of this initial hit-list of 1527 ligands was conducted by redocking with Glide and GOLD and scoring with their respective scoring functions and MM-GBSA.

To improve the likelihood of identifying actual active compounds, three sampling algorithms and four scoring functions were applied to the hit-list identified by Vina in a consensus based approach. This approach has frequently improved enrichment rates of active compounds in a virtual screening context, in comparison to the use of a single algorithm for docking and scoring [62,63,64]. The initial stage of refinement included redocking with Glide under standard precision and rescoring with its internal Glide GScore scoring function. In order to define a threshold binding score cut-off to prefilter hits, the crystallographic structure of 3V3M, with bound inhibitor ML188, was used. As most scoring functions cannot be compared between structures it is important to define a binding score cut-off based on a related experimental structure [57]. The inhibitor ML188 achieved a binding affinity estimation of −8.1 kcal/mol when bound to 3V3M. In an effort to reduce the numbers of false negatives this value was decreased to −7 kcal/mol. As a subsequent stage of hit-list refinement the identified hit compounds and 2ZU2 receptor were directly imported into GOLD for redocking and rescoring. A GOLDScore Fitness cut-off was defined as 60 and again used to prefilter hits [42].

At this stage the hit-list was refined to a point where the binding pose of each hit could be analyzed by manual inspection. The natural substrate of the 3CL^pro^ forms a hydrogen bond with His162/3 in the S_1_ pocket where the S_2_ pocket is filled by a hydrophobic moiety. Each ligand was therefore assessed with the Pose Filter script of Schrödinger and manually in Maestro to confirm that each ligand identified in the hit-list met these pharmacophoric constraints. These comprehensive filters of three sampling and scoring algorithms and pharmacophoric feature constraints provided us with a highly defined hit-list of 116 compounds. Assessing the free energy of binding with the highly advanced MM-GBSA scoring function would provide further confidence that the hits identified do in fact represent high-likelihood active compounds.

Rescoring the final hit-list with MM-GBSA identified a total of 22 ligands that passed all filters and pharmacophoric constraints for the 2ZU2 receptor. This included a total of three sampling algorithms and four scoring functions from Vina, Glide and GOLD and the highly advanced MM-GBSA, respectively, and two pharmacophoric constraints with a hydrogen bond acceptor in the S_1_ pocket and a hydrophobic moiety filling the S_2_ pocket. This consensus docking and scoring approach is in comparison to several studies, which identified novel lead compounds from a virtual screen, where only a single molecular docking algorithm was incorporated [65,66,67,68,69]. The comprehensive list of techniques used in this study heightens the probability that the ligands identified in the final hit-list do represent novel scaffolds of active molecules and with the high homology between the 3CL^pro^ of various human coronaviruses these compounds should exhibit a high potential for broad spectrum effects.

### 3.4. Pharmacophore Modeling

Varying techniques in drug design have led to the identification of different ligands from the same dataset and even identification of divergent scaffolds [70,71,72]. The use of multiple techniques therefore improves the probability of identifying a diverse set of lead compounds. The pharmacophore model was based largely on a single covalent inhibitor. This inhibitor was chosen as it possesses several favorable noncovalent features for the generation of a pharmacophore model. As general practice, several inhibitors are often used to generate a pharmacophore model where features that are frequently repeated are included in the pharmacophore [73]. For 3CL^pro^ these features are well documented [32,70,71,74] and the presence of several active ligands would only serve to replicate the prior knowledge. The essential features of the pharmacophore model included a hydrogen bond acceptor in the P_1_ position and a hydrophobic moiety in the P_2_ position. Non-essential features included a hydrogen bond donor and acceptor in the position to interact with Glu166, a hydrogen bond acceptor in the P_4_ pocket, a single hydrogen bond donor in the P_2_ and P_3_ pockets and an acceptor in the P_1′_ pocket. A total of four features, including the two essential features and any two of the non-essential features, within an excluded volume of 1 Å were required for a potential hit to satisfy the pharmacophore model.

Molecular Operating Environment (MOE) was used to generate the pharmacophore model and conduct the virtual screen against the entire ZINC Drugs-Now dataset. To ensure a primary hit-list of manageable size, only ligands satisfying the pharmacophore within an RMSD of 0.5 Å where filtered to the initial hit-list, which included approximately 25,000 compounds. Since MOE utilizes a united atom force field, it was necessary to add hydrogens with Open Babel. The protonation of the ligands generated several steric clashes within the active pocket of 3D23 and refine-only docking with Glide was therefore used to remove these clashes. As the pharmacophore model only filters compounds based on the presence of a hydrogen bond acceptor in the P_1_ position, and does not take into account distance and geometry essential for the formation of a hydrogen bond, the Pose Filter script of Schrödinger was again used to further refine the hit-list, which now contained 3569 hits. Scoring this hit-list with MM-GBSA further reduced its size to 15 compounds with the previously defined cut-off value.

### 3.5. Identification of Potential Broad Spectrum Inhibitors

The two virtual screening approaches, including molecular docking against 2ZU2, and pharmacophore modeling with the active inhibitor of 3D23, provided us with 22 and 15 hits respectively. Initial attempts to redock these hits into the individual binding pockets of the remaining four structures in order to identify potential broad spectrum inhibitors proved highly unsuccessful. Neither the binding mode nor binding affinity or free energy of binding could be replicated for any complex studied. This finding was in contradiction to our hypothesis that the high degree of homology between various active sites should allow for development of broad spectrum inhibitors.

The static nature of the receptor during docking did not allow for any degree of the induced-fit phenomena, which is commonly observed during ligand or substrate binding [75]. Although the residues within the active site of individual 3CL^pro^ are highly homologous, the orientation of their sidechains is variable between structures, which hinder ligand binding. In a dynamic, physiological system, these sidechains would accommodate ligand binding by the induced-fit phenomena, however in molecular docking this process is circumvented by the static nature of the receptor [76]. This was the sole reason for the inability to replicate the binding mode, seen in 2ZU2 and 3D23, in the remaining structures. The orientation of residue sidechains inserting into the active pocket were highly variable and the inability to replicate the dynamic movements of these sidechains during docking led to the highly variable and inefficient binding modes with decreased binding affinity.

To overcome this short fall of molecular docking, each receptor was aligned and ligands were superimposed over individual active sites using coordinates identified while docking to 2ZU2 or refine-only docking with 3D23. Residue sidechains resulting in a steric clash with the ligand were refined using the Prime module of Schrödinger, which replicates their dynamic, flexible movements allowing the sidechains to accommodate the ligand in the pocket. The ligand was then minimized within the pocket to remove any further steric clashes.

**Figure 2:**
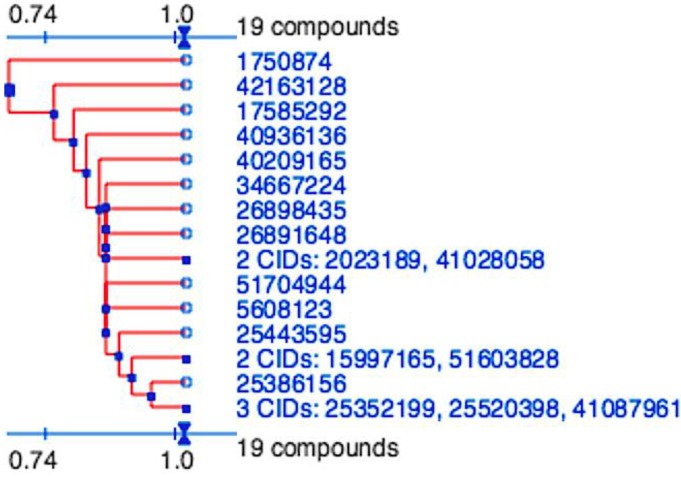
Division of the nineteen candidate ligands into fifteen distinct 3D clusters, as determined by the PubChem (https://pubchem.ncbi.nlm.nih.gov) structure-clustering web-service. 3D Tanimoto similarity scores were calculated from alignments generated using shape + feature and ten conformers per ligand. The dendogram was generated using similarity score of 1.04, which is recommended to achieve statistical significance at the 95% confidence interval.

The binding mode predicted using this approach was then further assessed using the pharmacophoric constraints of a hydrogen bond in the S_1_ and a hydrophobic moiety in the S_2_ pockets and scoring with MM-GBSA where the cut-off previously defined was again implemented. Of the 22 compounds identified in the hit-list of potential 2ZU2 binders, 15 compounds could replicate the interactions in the S_1_ and S_2_ pockets and fulfil the MM-GBSA cut-off (Table 1), where four of the compounds identified by pharmacophore modeling could fulfil these requirements (Table 2). This final list of 19 ligands represented 15 distinct clusters (Figure 2). Free energy of binding between a known active inhibitor, ML188, and respective coronavirus 3CL^pro^ is also shown in Table 1 and Table 2 for comparative purposes.

**Table 1:** Series of ligands identified by molecular docking with respective binding affinities and root-mean-square deviation (RMSD) between predicted poses from individual sampling algorithms. Initial virtual screen utilized 2ZU2 as the receptor. Binding affinity and mode was predicted by four scoring functions and three sampling algorithms respectively. Broad spectrum potential was assessed by scoring with molecular mechanics-generalized Born surface area (MM-GBSA).

ZINC ID	Binding Affinity in 2ZU2				RMSD Vina-Glide	RMSD Glide-GOLD	MM-GBSA of Remaining Structures			
	Vina	Glide	GOLD	MM-GBSA			3V3M	3D23	3TLO	OC43
27332786	−9.5	−7.43	73.75	−89.4	1.69	1.58	−100.8	−106.1	−95.8	−96.4
20130947	−9.5	−9.04	74.43	−83.6	0.03	0.045	−90.1	−96.0	−88.4	−87.7
15987063	−9.8	−8.34	68.43	−82.3	6.89	0.007	−91.0	−91.1	−79.3	−87.3
02466851	−9.5	−8.92	75.65	−100.3	7.14	0.01	−98.0	−104.3	−79.7	−89.8
12798320	−9.6	−8.18	72.81	−89.5	1.48	0.006	−83.0	−97.4	−79.7	−94.3
09477134	−9.6	−7.72	75.80	−86.0	4.09	0.005	−98.0	−88.4	−77.3	−84.1
32983195	−9.5	−7.02	65.44	−82.7	0.98	2.27	−81.6	−79.4	−73.1	−77.8
09104621	−9.8	−7.33	70.91	−83.3	1.20	0.002	−79.6	−100.6	−69.4	−87.7
02328322	−9.7	−8.53	75.37	−81.8	0.94	0.01	−89.2	−85.5	−73.8	−83.7
12550995	−9.5	−8.92	74.56	−81.4	1.62	0.01	−93.1	−84.1	−74.2	−78.7
12697660	−9.5	−7.68	75.08	−90.4	7.76	0.001	−94.1	−88.1	−62.4	−77.7
11783131	−9.6	−8.30	73.74	−84.0	6.74	0.01	−97.9	−80.1	−75.3	−87.7
12597223	−9.5	−9.00	68.90	−81.8	4.48	0.004	−76.7	−81.1	−68.7	−75.4
15999133	−9.7	−8.00	69.40	−81.9	6.22	0.003	−79.0	−97.1	−70.1	−91.5
35829976	−9.6	−8.33	70.23	−80.2	6.10	0.01	−70.0	−92.2	−60.9	−79.0
ML188	−7.9	−8.1	N/A	−85.0	0.244	N/A	−83.1	−85.2	−78.5	−85.3

**Table 2:** Free energy of binding as predicted by MM-GBSA for a series of ligands identified by pharmacophore modeling, with known inhibitor ML188 as a reference.

ZINC ID	MM-GBSA				
	3D23	2ZU2	3V3M	3TLO	OC43
09411012	−85.2	−84.6	−81.9	−77.5	−90.9
02426719	−103.0	−80.7	−85.5	−73.7	−109.3
02094118	−83.3	−90.8	−69.8	−82.8	−102.8
09346433	−92.5	−79.2	−81.9	−63.1	−103.4
ML188	−85.2	−77.4	−83.1	−78.5	−85.3

### 3.6. Molecular Dynamics

Molecular dynamics utilizes Newtonian physics to simulate atomic movements in a solvated system and remains the most accurate computational method for simulating protein-drug interactions. To confirm the findings seen in docking and pharmacophore modeling and validate the binding mode of representative hit-compounds, a 10 ns molecular dynamic simulation allowed for an assessment of non-bonded energies and hydrogen bonding networks between ligand and receptor and ligand stability via RMSD, radius of gyration and changes in solvent accessible surface area. As molecular dynamic simulations are extremely computer intensive one representative ligand identified from molecular docking and one from pharmacophore modeling was used. The ability of molecular dynamic simulations to replicate the binding conformation and ligand stability would verify that the identified hit-list does in fact represent likely actives.

#### 3.6.1. Candidate Inhibitor Identified by Molecular Docking, ZINC27332786

**Figure 3:**
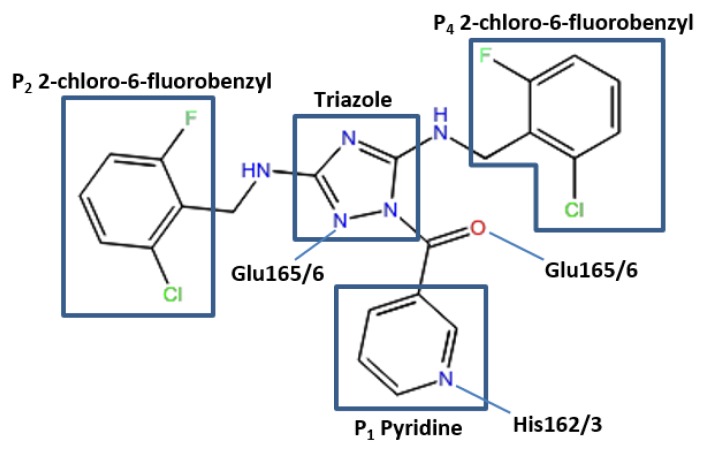
Pose of the ZINC27332786 ligand within the active site of the 3CL^pro^. Representation of elements mediating hydrogen bonds with respective residues and the P_1_ pyridine, hydrophobic P_2_ and P_4_ and a 1,2,4-triazole linker.

The conformation of the ZINC27332786 ligand, as identified by molecular docking results, indicated that it binds to the active site and forms similar key interactions throughout all five proteases studied. A pyridine filled the S_1_ pocket and formed a hydrogen bond between the pyridine nitrogen and imidazole Nε2 of His163. A 1,2,4-triazole formed a linker in the center of the structure and mediated the formation of a hydrogen bond between N2 and the backbone of Glu165/6, which formed a third hydrogen bond via the backbone carbonyl and ligand NH. OC43 3CL^pro^ exhibited a fourth hydrogen bond between N4 of the 1,2,4-triazole and Gln189. This residue is conserved in 3D23 and 3V3M structures, however it was orientated towards the solvent exposed exterior of the pocket. In 2ZU2 and 3TLO the position of Gln is replaced by Pro188/9, which therefore did not contribute to the formation of a bond. Two 2-chloro-6-fluorobenzyl rings also filled the hydrophobic S_2_ and S_4_ pockets (Figure 3).

Molecular dynamics studies indicated that for 3V3M all these interactions were highly conserved, forming an average of three bonds over the simulation with high occupancies between His163 and both bonds at Glu166. The remaining four structures exhibited similar profiles however with varying degrees of occupancy, with an average bond formation of 2.5 for 2ZU2, 1.7 for 3D23, 2.6 for 3TLO and 2.1 for OC43 3CL^pro^. All structures displayed high occupancy at both Glu166 positions, however a decreased occupancy at the His162/3 position. OC43 3CL^pro^ and 3D23 observed the lowest occupancy of 13.63% and 7.27% respectively and 2ZU2 and 3TLO displayed moderate occupancies of 25.30% and 41.05% respectively. The loss of hydrogen bond formation at this position was often caused by the pyridine ring orientating at a right angle to the His162/3 Nε2. With the exception of 3TLO all structures also formed hydrogen bonds with moderate frequency with Gln191/2. These bonds were predominantly formed via the backbone, however 2ZU2 was capable of forming with both the backbone and sidechain, where the sidechain exhibits a higher frequency. The bond formed between Glu189 and the N4 of the 1,2,4-triazole, as seen in docking results for OC43 3CL^pro^, exhibits a relatively low occupancy of 17.95%.

This indicated that results achieved from molecular dynamics were in relatively strong agreement with that of molecular docking, where all bonds were formed with high frequency, with the exception of the bond between the pyridine N and His162/3, which remained present, however at lower occupancy. The compound does, however, still provide an excellent scaffold for lead optimization with the substructures filling the S_2_, S_3_ and S_4_ subsites making extensive and stable interactions with surrounding residues including two hydrogen bonds between the central 1,2,4-triazole and NH and the backbone of Glu165/6. Further lead optimization may explore the replacement of the pyridine ring with the related lactam ring, which is known to mediate two hydrogen bonds, one between the ligand carbonyl and the His162/3 Nε2 and a second hydrogen bond between the lactam NH and Glu166 [46]. The presence of this second hydrogen bond and van der Waals interactions, mediated via ring substituents, may further stabilize the compound within the S_1_ pocket. A phenylalanine substituent or imidazole ring, has also shown to improve the inhibitory effects of peptidomimetic inhibitors [47,77].

**Figure 4:**
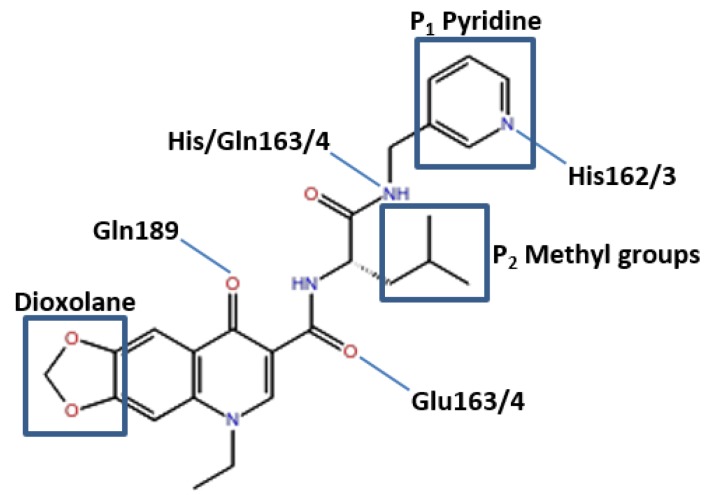
Pose of the ZINC09411012 ligand within the active site of the 3CL^pro^. Representation of elements mediating hydrogen bonds with respective residues and the P_1_ pyridine, hydrophobic P_2_ and solvent exposed dioxolane.

Analysis of ligand RMSD indicated that all structures deviated to some extent from their starting structures at the beginning of the simulation, but once in this position were stable and only minor fluctuations were observed from this point. Solvent accessible surface area of the ligand also remained consistent throughout the duration of the simulation and fluctuated within a range of 4.8 nm^2^–5.4 nm^2^ (Figure 4). Assessment of ligand radius of gyration also indicated that the compactness of the ligand did not alter greatly during the 10 ns simulation and fluctuated within 4.6 Å and 4.9 Å for all structures. This again indicated that the ligand is stable within the pocket and did not dissociate into surrounding solvent.

#### 3.6.2. Candidate Inhibitor Identified by Pharmacophore Modeling, ZINC09411012

**Figure 5:**
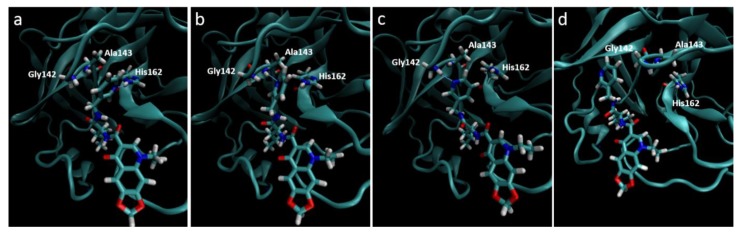
Destabilization of ZINC09411012 from the 2ZU2 active site. (**a**) Starting conformation where the pyridine fills the S_1_ pocket and forms a hydrogen bond with His162; (**b**) Initial destabilization resulting in the break of the hydrogen bond with His162 and subsequent formation with Ala143; (**c**) Further destabilization resulting a new hydrogen bond with Gly142; (**d**) Complete loss of orientation.

The 3CL^pro^ structures with bound inhibitor identified by pharmacophore modeling again indicated conserved interactions between the receptor and ligand. As with the previous compound a pyridine ring fills the S_1_ pocket and formed a hydrogen bond with the imidazole Nε2 of His162/3. A backbone carbonyl of His164 for 3V3M formed a hydrogen bond with a ligand NH. This residue was replaced by a Gln in all remaining structures, however a backbone carbonyl of these two residues were perfectly aligned thereby allowing the formation of the hydrogen bond in all structures. The backbone NH of Glu165/6 again formed a hydrogen bond with a ligand carbonyl. A fourth hydrogen bond is observed in 3V3M, 3D23 and OC43 3CL^pro^ between the sidechain of Gln189 and a ligand carbonyl. The position of this Gln in 2ZU2 and 3TLO was not aligned structurally to 3V3M, 3D23 or OC43 3CL^pro^, with structural superimposition showing a Pro in this position accounting for the loss of the hydrogen bond in 2ZU2 and 3TLO. Two methyl groups insert into the deep S_2_ pocket and made extensive hydrophobic interactions with surrounding residues. A potential problematic feature of this ligand was a solvent exposed 1,3-dioxolane, which made few interactions with surrounding residues. Further lead optimization steps may explore the removal of this substituent to ascertain any structure-activity relationship (Figure 5).

Molecular dynamics trajectories again showed a strong correlation with that seen in our pharmacophore models, with the exception of the 2ZU2-ZINC09411012 complex. In this structure the pyridine ring gradually dissociated out of the S_1_ pocket at around 4 ns followed by almost complete dissociation of the ligand from the active site, where the 1,3-dioxolane became completely solvent exposed. The two methyl groups that filled the hydrophobic S_2_ pocket remained the only substructures of the ligand to maintain any structural conformation in the active subsite, indicating the importance of this group in ligand stability. The orientation of all S_1_ subsite residues including His162, Phe139, Glu165, His171 and Tyr160 remained unaltered and therefore could not attribute to this loss of stability. The movement may most likely be attributed to by an initial minor loss of stability caused by the movement of Ala143 closer to the pyridine ring, with subsequent bond formation between the Ala backbone and pyridine N. This movement destabilized the pyridine ring, which subsequently formed a new hydrogen bond with Gly142. At this point the pyridine had almost completely lost its orientation in the S_1_ pocket, which resulted in further loss of the van der Waals interactions leading to complete dissociation from the pocket (Figure 5). This movement also corresponded to a gradually increasing RMSD, assuming few stable conformations, indicative of an unstable ligand.

The position of Ala143 in 2ZU2 is transformed to Ser in 3V3M, 3D23 and OC43 3CL^pro^, suggesting that the hydrophobic sidechain of the Ala in 2ZU2 may have promoted the movement towards the pyridine, encouraging rotation of the nitrogen towards the Ala backbone. The orientation of the pyridine ring in 3TLO is somewhat stabilized by a bifurcated interaction with the sidechain of Glu166 which promoted the movement of the pyridine N away from Ala143 without disrupting the interaction with His162. Lead optimization steps may therefore preferentially incorporate a core structure that prevents rotation of the pyridine by resulting steric clashes. As mentioned previously, the exploration of a phenylalanine substituent, imidazole or lactam ring in this position may also contribute positively to the stability of the ligand within the S_1_ pocket [46,47,77].

The remaining structures did however show stability of the ligand within the active pocket, where the occupancy of the hydrogen bond with the Nε2 of His162/3 ranged from 64% for 3TLO, 75% for OC43 3CL^pro^, 83% for 3V3M and 89% for 3D23. Interactions with the Glu165/6 were also highly conserved with a frequency of 86% for 3TLO, 91% for OC43 3CL^pro^ and 3V3M and 94% for 3D23. The position of 164, occupied by a His in 3V3M and a Gln in the remaining structures, showed varying degrees of bond formation, with the presence of the His in 3V3M resulting in a significant reduction in bond formation. Gln showed moderate, 41% for 3D23, to high, 88% for 3TLO, occupancy with OC43 3CL^pro^ having an occupancy of 61%. The sidechain of Gln189 was shown to form a hydrogen bond with the ligand in 3V3M, 3D23 and OC43 3CL^pro^, however this residue was highly solvent exposed and therefore resulted in a high degree of flexibility of the sidechain, which should hinder the formation of this bond. For 3D23 and OC43 3CL^pro^ this was observed, where the frequency of bond formation was 27% and 6% respectively. In 3V3M the ligand was however able to stabilize the sidechain to maintain the formation of the bond with a high degree of frequency at 73%.

Ligand RMSD again showed similar profiles to the previously described complexes, where initially a large movement was observed followed by subsequent stabilization of the ligand within the active site. Structures, including 3V3M and 3D23, observed significant fluctuations within their stable conformation which was largely attributed to by the presence of the solvent exposed dioxolane moiety, which had few features and interactions to stabilize it within the pocket. As this structure contributed few interactions, future studies may explore the removal of this substituent to ascertain its effects on stability. Radius of gyration again confirmed RMSD results where all structures fluctuate between 4.7 Å and 5.0 Å, with the exception of 2ZU2 which deterred greatly at approximately 6 ns that signified the point at which the pyridine ring completely dissociated from the S_1_ pocket. This however did not result in an increase in solvent accessible surface area where all structures fluctuated between 3.8 nm^2^ and 4.4 nm^2^. With the exception of 2ZU2, these structures confirmed, with great confidence, the interactions and conformations observed within our rigid pharmacophore model. The representative ligand identified by molecular docking was also able to largely fulfil the interactions and orientations observed.

## 4. Discussion

Lead compounds that have shown promise as potential inhibitors of the 3CL^pro^ of coronaviruses are largely represented by peptidomimetic compounds that covalently alter the thiolate anion of the active Cys residue [19]. These compounds are generally large and have a high likelihood of off target effects through covalent modification and, as yet, there remains no commercially available molecular entity for the treatment of coronaviruses [14]. Few studies have focused on the development of broad spectrum, noncovalent inhibitors and, to our knowledge, none have aimed to prioritize the identification of broad spectrum lead compounds that inhibit the 3CL^pro^ related to the coronaviruses that most frequently affect humans, namely 229E, OC43, NL63 and HKU1. For this reason, there is insufficient knowledge surrounding the structure of broad spectrum inhibitors targeting this family of proteases. This study, focusing on these and the coronavirus associated with SARS, provides valuable insight into the structure of ligand scaffolds of potential noncovalent inhibitors.

Different techniques used in virtual screening have led to the identification of a diverse set of lead compounds, where one technique is able to identify compounds the other may have missed [70,71]. For this reason, we used a joint docking and pharmacophore modeling based virtual screening strategy to identify a highly defined list of potential lead compounds. Molecular docking and pharmacophore modeling are extremely useful tools in the design and identification of novel lead compounds and have relatively low infrastructure and fiscal dependence. Its importance in the pharmaceutical industry cannot be overlooked even though it does have several shortcomings. Fundamental flaws in these structure-based techniques include the static nature of the receptor and the implicit solvent models used for virtual screening. Molecular dynamics is the most advanced *in silico* tool, which allows us to fill this void by incorporating Newtonian physics to simulate the dynamic movements of proteins in an explicit solvent model thereby replicating the native environment of the protein. Molecular dynamic simulations also provide a mechanistic explanation for any ligand inactivity, which would be impossible to identify with *in vitro* techniques. In this study, it has identified the key feature responsible for the destabilization of the ZINC09411012 ligand within the 2ZU2 binding pocket and thereby allowed us to identify which substituents should be replaced in further rounds of lead optimization. As ligand inactivity may be a result of a single misplaced substituent this mechanistic view point provides extensive information for future lead optimization.

The essential pharmacophoric constraints, including an acceptor in the P_1_ and hydrophobic moiety in P_2_, used in both docking and pharmacophore modeling drug discovery methods would be expected to generate a hit-list with similar scaffolds. The final hit-list, containing a total of 19 ligands (15 from molecular docking and four identified by pharmacophore modeling), however were highly divergent and represented 15 distinct clusters. These were determined using the PubChem (https://pubchem.ncbi.nlm.nih.gov) structure clustering web-service with shape + feature 3D similarity and recommended parameters for achieving statistical significance at the 95% confidence interval (Figure 5). 3D similarity calculation, using shape-only and feature-only, yielded similar results (data not shown). As all cluster members were already preselected for features to fill the P_1_ and P_2_ pockets, have similar estimated binding affinities and the structure clusters are significantly dissimilar, they represent 15 valuable scaffolds that can be further experimentally validated and to be used as a basis for future lead optimization and anti-coronaviral inhibitor discovery experiments.

In our simulations of potential inhibitors, previously identified by structure and ligand-based drug design techniques including molecular docking and pharmacophore modeling, we could largely replicate all interactions observed in our static models. The pyridine ring that fills the S_1_ pocket in 3V3M is stable in both modelled ligand-protein complexes and with the known inhibitor, ML188, it is evident that this feature contributes positively to antiviral effects of the inhibitor [19]. However, with the remaining 3CL^pro^, several complexes showed instability of the pyridine ring within the S_1_ pocket. We therefore propose that future lead optimization explores the effects of incorporating a lactam ring in this position as it is known to better replicate the interactions of the natural substrate and is also effective against the SARS-CoV 3CL^pro^ [46]. A phenylalanine substituent and an imidazole ring have also been shown to be efficacious in this P_1_ position [47,77]. The replication of the majority of interactions that were observed in the static structure-based models for ZINC27332786, from molecular docking, and ZINC09411012, from pharmacophore modeling, suggests that the structure-based techniques were correct in identifying the key interactions and orientation of the ligand within the pocket. We can therefore confidently assume that the remaining ligands identified in the structure-based techniques are, in fact, in their correct conformation and do represent promising lead compounds.

As the second generation of lead discovery focusing on non-peptidic, noncovalent inhibitors is still largely in its infancy, future *in vitro* activity assays of the ligands identified in this study will provide vital information on novel scaffolds for lead optimization. The diverse and comprehensive approach of hit-list refinement also ensures a high likelihood of the presence of bioactives within the list. This study provides a promising start in the pursuit of active lead compounds for the broad spectrum inhibition of the 3CL^pro^ associated with human coronaviruses. These results strongly support further *in vitro* testing of this highly refined hit-list. Activity data will provide valuable information on the structure of bioactive scaffolds and could represent the next generation of small lead molecules. In conclusion, in the drug-design sphere, a variety of computational approaches have been used at the various stages of the drug-design process in support of the experimental techniques. Structure-based and ligand-based drug design approaches have developed into valuable drug discovery tools [78], owing to their versatility and synergistic character [79]. Importantly, however, predictions from virtual screening methods are not meant to replace experimental affinity determination, but can complement the experimental methods by producing an enriched subset of a large chemical database, with an increased possibility of the compounds actually binding to the drug target of interest [80,81]. Therefore, since past research has shown that many predicted drug candidates fail to prove effective *in vivo* the experimental validation of the identified 19 ligands reported in this manuscript is essential. 
